# Migraine treatment: *quo vadis*? Real-world data study (2015–2022) in Spain

**DOI:** 10.1186/s12883-024-03600-8

**Published:** 2024-04-02

**Authors:** Patricia Pozo-Rosich, Mafalda Carmo, Alejandro Muñiz, Beatriz Armada, Carlota Moya-Alarcón, Julio Pascual

**Affiliations:** 1 Neurology Department, Headache Unit, Hospital Universitari Vall d’Hebron, 119-129 Passeig de la Vall d’Hebron, Barcelona, 08035 Spain; 2 Headache Research Group, Vall d’Hebron Research Institute (VHIR), Universitat Autònoma de Barcelona, Barcelona, Spain; 3 IQVIA, Barcelona, Spain; 4 IQVIA, Madrid, Spain; 5 Pfizer, Madrid, Spain; 6 Service of Neurology, University Hospital Marqués de Valdecilla, Universidad de Cantabria and Valdecilla Biomedical Research Institute (IDIVAL), Santander, Cantabria Spain

**Keywords:** Migraine, Management, Spain, Epidemiology, Acute, Preventive, Diagnosis, CGRP, Triptan, Patterns

## Abstract

**Background:**

Migraine is a leading cause of disability, estimated to affect one-in-ten people in Spain. This study aimed to describe the management of migraine in Spain and identify improvement areas.

**Methods:**

Non-interventional, retrospective, cross-sectional cohort study conducted using an electronic medical records database covering visits to public healthcare providers for 3% of the Spanish population. Patients with a migraine diagnosis (ICD-9 346) between 01/2015 and 04/2022 were included, as well as their demographic and clinical characteristics, prescribed migraine treatments and the specialty of the prescribing physicians.

**Results:**

The database included 61,204 patients diagnosed with migraine. A migraine treatment had been prescribed to 50.6% of patients over the last 24 months (only acute to 69.5%, both acute and preventive to 24.2%, and only preventive to 6.3%). The most frequently prescribed treatments were NSAIDs (56.3%), triptans (44.1%) and analgesics (28.9%). Antidepressants were the most common preventive treatment (prescribed to 17.9% of all treated patients and 58.7% of those treated with a preventive medication), and anti-CGRP monoclonal antibodies the least prescribed (1.7%; 5.7%). In 13.4% of cases, preventive medications were the first treatment: alone in 5.8% of cases and together with an acute medication in 7.6%. A fifth of patients who were initially prescribed with only acute treatment were later prescribed a preventive medication (20.7%). On average, it took 29.4 months for this change to occur. Two-thirds of patients started their preventive treatment in primary care (64.2%). The percentage of patients treated by a neurologist increased with the number of received preventive medications. However, 28.8% of patients who had already been prescribed five or more distinct preventive treatments were not treated by a neurologist. Migraine patients had between 1.2- and 2.2-times higher prevalence of comorbidities than the general population, age-gender adjusted.

**Conclusions:**

Our study emphasizes the need for improved management of migraine in Spain to reduce the risk of chronification and improve patient outcomes. More training and coordination across healthcare professionals is necessary to recognize and address risk factors for migraine progression, including multiple associated comorbidities and several lines of treatment, and to provide personalized treatment plans that address the complex nature of the condition.

**Supplementary Information:**

The online version contains supplementary material available at 10.1186/s12883-024-03600-8.

## Background

Migraine is a debilitating condition affecting over 14% of the global population [1]. It is the second leading cause of disability and the first among women aged between 15 and 49 years old [2]. The condition is typically classified as either episodic migraine (EM) or chronic migraine (CM), based on the frequency of headache days per month, with CM defined as having ≥ 15 headache days per month, eight of which should have migraine characteristics [3–5]. Both forms of migraine are highly debilitating, and can negatively impact the patients’ quality of life, ability to work, and generate substantial direct and indirect economic costs [5–10]. 

Progression from EM to CM occurs at a rate of 2.5% per year and is influenced by various factors, including migraine disease characteristics, treatment-related factors, comorbidities, lifestyle/exogenous factors, and demographic factors [5, 6, 11, 12]. Preventing migraine progression involves managing comorbidities, promoting healthy lifestyle habits, and optimizing acute and preventive treatment [12]. Preventive treatment is used to reduce the frequency, duration and severity of migraine attacks, while acute treatment is used to manage symptoms from migraine attacks [13–15]. Acute therapies include migraine-specific (e.g. triptans and ergot alkaloids) and non-migraine specific agents (e.g. analgesics and nonsteroidal anti-inflammatory drugs [NSAIDs]) [5, 16, 17]. Research indicates that optimizing acute headache medication is an effective strategy in reducing the risk of migraine progression and the likelihood of developing medication overuse headache (MOH) [12]. 

The use of preventive treatment can reduce the risk of migraine progression - by reducing migraine attacks and limiting acute medication use-, and increase patients’ transition rates from CM to EM, but concerns over the lack of long-term efficacy and tolerability from conventional migraine treatments often lead to treatment interruption, especially in CM [18–28]. Treatment discontinuation rates are estimated at 40.8% for CM and 24.0% for EM [29]. Less than 10% of CM patients stop their preventive medication because of an improvement: the majority stop due to a lack of efficacy (EM 36.8-47.6%, CM 39.2-48.2%) and lack of tolerability (EM 34.8-49.0%; CM 34.2-53.2%) [29]. 

Anti-calcitonin gene-related peptide (CGRP) monoclonal antibodies (mAbs), are novel therapies which have been approved in Spain for the treatment of migraine and have a good efficacy and tolerability [13, 30–32], decreasing acute headache medication use and reducing monthly headache days even in migraine patients with medication overuse headache [18]. By April 2022, three anti-CGRP mAbs were reimbursed in Spain for preventive migraine treatment for adults with ≥ 8 migraine days a month and ≥ 3 prior treatment failures – necessarily including botulinum toxin type A [BoNT/A] for CM [33]. Anti-CGRP are considered to be a successful treatment strategy and Spanish Scientific Societies recommend that access to these novel treatments is improved [34, 35]. 

Around one-in-ten people report suffering from migraine in Spain, of whom 70–90% are diagnosed with migraine and 10–30% have undiagnosed migraine [36, 37]. In Spain, as in other European countries, migraine is not only underdiagnosed but also undertreated [6, 38, 39]. Migraine can also be difficult-to-treat. Some patients go through different preventive medications – due to prior treatment failure – raising concerns on medication overuse [39]. Not receiving an appropriate treatment, acute and preventive, at the right time, might help increase resistance and refractoriness [40, 41]. 

To improve the management of migraine - and reduce its burden - one must start by understanding the patients’ profile and pathways in clinical practice. This study aims to contribute with real-world-evidence to help improve the management of migraine in Spain.

## Methods

### Study design

This was a non-interventional, retrospective, cross-sectional cohort study conducted using IQVIA’s Electronic Medical Records (EMR) longitudinal database, which covers 3% of the Spanish population. Patients diagnosed with episodic and chronic migraine by National Health Service (NHS) physicians between January 2015 and April 2022 were identified. The primary objective was to understand the migraine-associated treatments prescribed to patients with a migraine diagnosis. The secondary objectives were to (1) describe the characteristics of patients with migraine, (2) describe the presence of coexisting diagnosis with migraine (comorbidities), (3) identify the healthcare professionals who diagnose migraine and who prescribe migraine-associated treatments.

### Database

IQVIA’s EMR longitudinal database represents the entire public healthcare infrastructure (outpatient settings) of three distinct regions in Spain, comprising approximately 1.2 million patients - representing 3% of the Spanish population; and 1,450 general practitioners (GPs) and 66 neurologists – corresponding to 4.1% and 3.2% of the respective physician’s universe, in addition to other specialists. Due to the sensitive nature of this information and the presence of confidentiality agreements with both the database provider and the respective regions, we are unable to disclose the specific regions included in the database. The database contains anonymized data from all outpatient visits to the NHS (i.e., hospitalizations are not included) for these health areas, using a unique patient identifier through the NHS. The information collected in the database is provided by the regions themselves and reflects the real-world coding practices in Spain. The following variables were extracted for this study: age, gender, received diagnoses and respective date, prescriptions (by Anatomical Therapeutic Chemical [ATC] code, strength, daily dose, date, and duration), and the specialty of the prescribing physicians.

### Study definitions

#### Migraine cases

Migraine patients were defined as those in the EMR database who received at least a diagnosis for migraine during the study period (i.e., physician-diagnosed patients), defined as those coded with International Classification of Diseases 9th Revision (ICD-9) 346. Due to limitations from the database, cases were not segmented into CM and EM nor according to the patients’ headache frequency.

#### Headache cases

People suffering from headaches were separately identified, defined as those receiving at least an ICD-9 headache diagnosis 784.0, 307.81 or 339 and being treated with products used for migraine **(Table S2)**. These patients were not included in the studied population but were quantified as potential misdiagnosed migraine cases.

#### Relevant comorbidities

Comorbidities were defined as the active diagnoses associated to the population in the EMR during the analyzed period. Ten groups of comorbidities were analyzed: cardiovascular, stroke, digestive, metabolic, neurologic, neuropsychiatric, pain, respiratory, rheumatologic, and sleep conditions. The detailed list of analyzed comorbidities, along with the respective ICD-9 codes, is detailed in Supplementary Materials (Table S1).

#### Migraine treatments

Treatments were selected based on national guidelines and classified as preventive or acute [42, 43]. Ergot alkaloids, antiemetics, opioids, serotonin agonists (triptans), analgesics, and NSAIDs were classified as acute migraine treatments. Antivertigo preparations (including flunarizine), anti-CGRP mAbs, antiepileptics, antidepressants, and beta blocking agents were classified as preventive. Only prescriptions in which migraine was the selected diagnosis were included. Benzodiazepines are analyzed as a relevant migraine associated treatment but not as a migraine treatment per se. The complete list of molecules included in the analysis is detailed in Supplementary Materials (Table S2). Treatments without prescription or administrated at the hospital were not included, as they are not registered in the EMR database. As a result, BoNT/A is not present in the study. Prescribed anti-CGRP mAbs are captured by the database. At the time of the study, three were reimbursed by the NHS in Spain. Erenumab and galcanezumab were marketed in November 2019 and fremanezumab in August 2020 [33]. 

#### Lines of treatment

The study included patients with migraine between January 2015 and April 2022. For each patient, the index date was defined as the date of initiation of the first migraine treatment. Patients were followed from the index date until the end of the study period (April 2022). A pre-index date was established from January 2013 to December 2014 - or before when available, to ensure that patients had not been treated before.

A patient’s line of treatment starts with the first prescription for migraine, within the list of preventive or acute medications included in the study (Table S2). As illustrated in Figure S1, a change in line of treatment may be due to a switch - when the full treatment is changed; an add-on - when there is no treatment interruption, but one or more medications are added; or a drop-of - when there is no treatment interruption, but one or more medications are stopped.

### Selected patient cohorts

Six cohorts were analyzed, namely:

A. **Cohort of patients treated in the last two years**: Included only patients treated between May 2020 and April 2022. All their respective treatments during that period were included. This cohort was analyzed as it corresponds to the most recent data obtainable and in which anti-CGRP mAbs were already available.
B. **Cohort of patients who initiated any preventive or acute treatment between 01/2015 and 12/2016**: Included only patients who initiated treatment with a preventive or acute treatment between January 2015 and December 2016. All their migraine treatment prescriptions until the end of the study period (April 2022) were included. This cohort was analyzed to enable a longitudinal analysis of the pharmacologic treatment and disease management pathways through time.
C. **Cohort of patients who initiated treatment with triptans between 01/2015 and 12/2016**: Included only patients who initiated treatment with triptans between January 2015 and December 2016. Patients were then followed until April 2022. Only triptans prescriptions were considered.
D. **Cohort of patients who initiated treatment with a preventive medication between 01/2015 and 12/2016**: Included only patients who initiated treatment with preventive treatment between January 2015 and December 2016. Patients were then followed until April 2022. Only preventive prescriptions were considered.
E. **Cohort of patients who initiated any migraine treatment between 01/2015 and 12/2016 and were eventually prescribed anti-CGRP mAbs**: Included only patients who initiated treatment with a preventive or acute treatment between January 2015 and December 2016 and who were prescribed an anti-CGRP mAbs at some point until April 2022. All their migraine treatment prescriptions until the end of the study period (April 2022) were included.
F. **Cohort of patients who were prescribed anti-CGRP mAbs**: Included only patients who were prescribed an anti-CGRP mAbs at some point until April 2022. All acute and preventive treatments prescribed to these patients between January 2015 and April 2022 were included.

### Statistical analysis

Subjects included in IQVIA’s EMR database are representative of the Spanish population, by age and gender distribution [44]. The statistical analysis was performed on the selected population in the EMR database, based on criteria established in the study protocol, as approved by the Ethics Committee of the Hospital Clinic of Barcelona (HCB/2022/1286). For continuous variables, descriptive statistics are presented as mean or median. For categorical variables, statistics are presented as absolute and relative frequencies. The chi-squared test was used to compare the rate of comorbidities in both the group of patients diagnosed with migraine and the group of patients on treatment for migraine over the past 24 months with a stratified random sample of the general population in the database, matching the age and gender distribution of the patients diagnosed with migraine.

### Ethical considerations

The study was conducted following the ethical principles of the Declaration of Helsinki and the local regulation, including privacy laws. The protocol was classified by the Agency of Medicines and Medical Devices (AEMPS) as an observational study and approved by the Ethics Committee of the Hospital Clinic of Barcelona (HCB/2022/1286). Informed consent to participate was waived by the same ethics committee that approved the study (Ethics Committee of Hospital Clinic of Barcelona).

## Results

### Demographic and clinical characteristics of the studied population

**Fig. 1:**
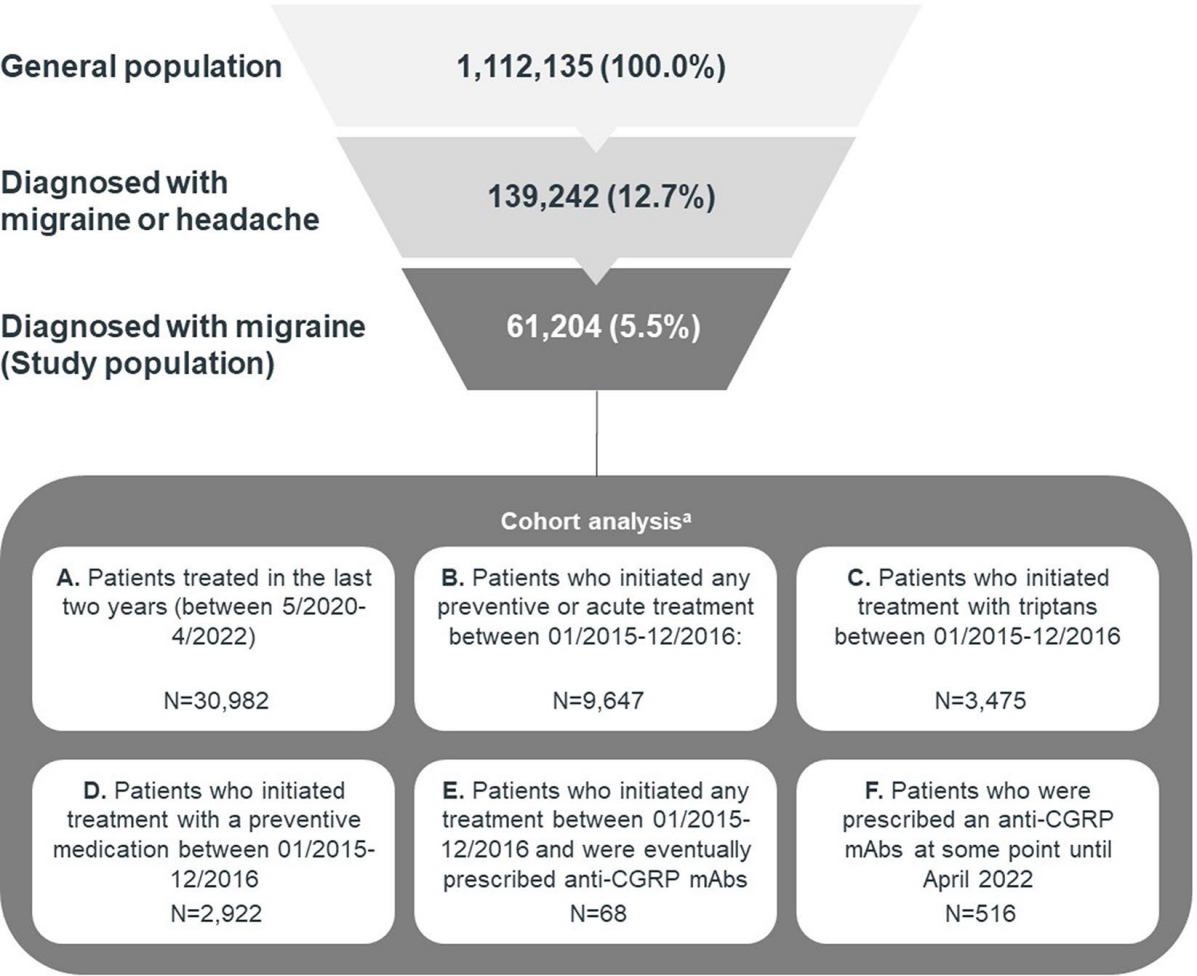
Patients from the database included in the analysis. * The cohorts are not mutually exclusive

From May 2021 to April 2022, there were 61,204 people with physician-diagnosed migraine in the database, corresponding to 5.5% of the covered population (1,112,135 people). Half of the patients (30,982/61,204; 50.6%) had received at least a preventive or acute treatment over the last 24 months (Fig. 1). The incidence of diagnosed migraine increased at a mean annual growth rate of 5.1% over the study period.

Table 1 details the demographic and clinical characteristics for: (a) patients with migraine; (b) patients with migraine and treated for migraine within the past 24 months; (c) overall population in the EMR database; (d) an age and gender adjusted sample of the general population, to match the patients with migraine. Women accounted for 77.7% of the diagnosed migraine cases. Regardless of gender, nearly three out of four migraine patients were aged between 19 and 59 years old. Migraine patients presented a higher prevalence of chronic comorbidities than the general population in the database, even after adjusting for age and gender (Table 1). The differences were statistically significant (*P* < 0.001) for all analyzed groups of comorbidities. Presence of chronic pain disorders (50.8%), metabolic (38.6%), neuropsychiatric (35.0%), digestive (25.4%) and cardiovascular (24.5%) diseases were the most frequently registered comorbidities in patients with migraine (Table 1).

**Table 1:** Demographic and clinical characteristics of physician-diagnosed migraine patients, physician-diagnosed migraine patients treated for migraine within the past 24 months, and overall population covered by the electronic medical records database from May 2021 to April 2022

Characteristics	Patients diagnosed with migraine (*n* = 61,204)	Patients diagnosed with migraine on treatment (*n* = 30,982)	General population (*n* = 1,112,135)	Age and gender adjusted sample of the general population (*n* = 100,002)
**Gender, n (%)**				
Female	47,585 (77.7)	25,177 (81.3)^***^	577,197 (51.9)	77,750 (77.7)
Male	13,618 (22.3)	5,804 (18.7)^***^	534,937 (48.1)	22,251 (22.3)
**Age, n (%)**				
**Female**				
< 12 years old	66 (0.1)	42 (0.2)^***^	54,833 (9.5)	108 (0.1)
12–18 years old	778 (1.6)	481 (1.9)^***^	38,672 (6.7)	1,271 (1.6)
19–29 years old	4,463 (9.4)	2,314 (9.2)^***^	68,109 (11.8)	7,293 (9.4)
30–39 years old	7,826 (16.4)	4,031 (16)^***^	79,653 (13.8)	12,788 (16.4)
40–49 years old	11,630 (24.4)	6,636 (26.4)^***^	94,083 (16.3)	19,002 (24.4)
50–59 years old	10,715 (22.5)	6,062 (24.1)^***^	84,847 (14.7)	17,507 (22.5)
≥ 60 years old	12,104 (25.4)	5,609 (22.3)^***^	156,997 (27.2)	19,777 (25.4)
**Male**				
< 12 years old	58 (0.4)	39 (0.7)	58,307 (10.9)	96 (0.4)
12–18 years old	486 (3.6)	247 (4.3)	40,120 (7.5)	794 (3.6)
19–29 years old	1,642 (12.1)	585 (10.1)	62,587 (11.7)	2,684 (12.1)
30–39 years old	2,171 (15.9)	871 (15)	71,146 (13.3)	3,548 (15.9)
40–49 years old	3,234 (23.8)	1,462 (25.2)	92,009 (17.2)	5,285 (23.8)
50–59 years old	2,863 (21)	1,340 (23.1)	81,310 (15.2)	4,678 (21)
≥ 60 years old	3,160 (23.2)	1,257 (21.7)	129,455 (24.2)	5,163 (23.2)
**Comorbidities, n (%)**				
Cardiovascular	14,995 (24.5)^***^	7,250 (23.4)^***^	222,427 (20)	21,673 (21.7)
Acute pulmonary heart diseases	184 (0.3)	93 (0.3)	3,336 (0.3)	339 (0.3)
Chronic cardiac rheumatic disease	184 (0.3)	94 (0.3)	3,558 (0.3)	353 (0.4)
Arterial hypertension	11,629 (19)^***^	5,670 (18.3)^***^	173,492 (15.6)	17,025 (17)
Ischemic disease (AMI/AP)	1,408 (2.3)	527 (1.7)^***^	28,915 (2.6)	2,197 (2.2)
Other CV diseases	3,856 (6.3)^***^	1,673 (5,0.4)	62,279 (5.6)	5,678 (5.7)
Peripheral CV Disease	1,346 (2.2)^***^	651 (2.1)^***^	18,906 (1.7)	1,745 (1.7)
Stroke	1,653 (2.7)^***^	806 (2.6)^***^	23,021 (2.1)	2,063 (2.1)
Digestive	15,546 (25.4)^***^	8,582 (27.7)^***^	164,596 (14.8)	16,783 (16.8)
Gastrointestinal diseases 1^b^	11,323 (18.5)^***^	6,506 (21)^***^	108,989 (9.8)	12,191 (12.2)
Gastrointestinal diseases 2^c^	5,692 (9.3)^***^	2,881 (9.3)^***^	66,727 (6)	5,943 (5.9)
Metabolic	23,625 (38.6)^***^	11,866 (38.3)^***^	311,397 (28)	33,079 (33.1)
Dyslipidaemia	15,668 (25.6)^***^	7,807 (25.2)^***^	212,417 (19.1)	21,787 (21.8)
Obesity	7,773 (12.7) ^***^	3,873 (12.5)^***^	96,755 (8.7)	10,372 (10.4)
Diabetes (Type 1 / Type 2)	4,407 (7.2)^***^	1,983 (6.4)^***^	84,522 (7.6)	7,690 (7.7)
Hypothyroidism	4,774 (7.8)^***^	2,510 (8.1)^***^	44,485 (4)	6,342 (6.3)
Neurologic	796 (1.3)^***^	434 (1.4)^***^	10,009 (0.9)	923 (0.9)
Epilepsy	796 (1.3)^***^	434 (1.4)^***^	10,009 (0.9)	923 (0.9)
Neuropsychiatric	21,421 (35)^***^	12,703 (41)^***^	180,165 (16.2)	21,881 (21.9)
Anxiety, Dissociative and Somatoform Disorders	17,198 (28.1)^***^	9,666 (31.2)^***^	144,537 (13)	18,229 (18.2)
Bipolar Disorder	734 (1.2)^***^	434 (1.4)^***^	6,672 (0.6)	800 (0.8)
Depression	3,489 (5.7)^***^	2,014 (6.5)^***^	30,027 (2.7)	3,701 (3.7)
Drug abuse	122 (0.2)^***^	62 (0.2)^***^	1,111 (0.1)	97 (0.1)
Drug dependency	190 (0.3)^***^	93 (0.3)^***^	2,223 (0.2)	181 (0.2)
Other syndromes	3,795 (6.2)^***^	3,563 (11.5)^***^	15,569 (1.4)	2,119 (2.1)
Stress syndromes	61 (0.1)^*^	31 (0.1)	1,118 (0.1)	68 (0.1)
ADHD	245 (0.4)^***^	155 (0.5)^***^	6,672 (0.6)	213 (0.2)
Pain	31,092 (50.8)^***^	16,482 (53.2)^***^	324,743 (29.2)	37,466 (37.5)
Chronic Pain, Fibromyalgia, Generalised Pain	5,202 (8.5)^***^	2,912 (9.4)^***^	50,046 (4.5)	5,794 (5.8)
Dorsopathies	23,441 (38.3)^***^	12,672 (40.9)^***^	227,987 (20.5)	26,836 (26.8)
Chronic Fatigue Syndrome	6 (0)	3 (0)	222 (0)	3 (0)
Disorders of the peripheral nervous system	7,222 (11.8)^***^	3,904 (12.6)^***^	58,942 (5.3)	7,195 (7.2)
Osteoarthrosis and related disorders	7,528 (12.3)^***^	3,532 (11.4)^***^	87,858 (7.9)	10,101 (10.1)
Temporomandibular joint disorders	612 (1)^***^	341 (1.1)^***^	4,448 (0.4)	539 (0.5)
Respiratory	7,228 (11.8)^***^	3,718 (12)^***^	83,410 (7.5)	7,937 (7.9)
Asthma / COPD	6,365 (10.4)^***^	3,315 (10.7)^***^	77,849 (7)	7,265 (7.3)
Sinusitis	979 (1.6)^***^	527 (1.7)^***^	6,672 (0.6)	797 (0.8)
Rheumatologic	490 (0.8)^*^	248 (0.8)	5,560 (0.5)	695 (0.7)
Arthritis	490 (0.8)^*^	248 (0.8)	5,560 (0.5)	695 (0.7)
Sleep	6,916 (11.3)^***^	3,966 (12.8)^***^	67,840 (6.1)	7,704 (7.7)

ADHD, Attention-deficit/hyperactivity disorder; AMI, Acute myocardial infarction; AP, Angina pectoris; COPD, Chronic obstructive pulmonary disease; CV, cardiovascular

a. Adjusted by age and gender for the general population

b. Includes ICD-9 diagnoses codes 530–539 and 569

c. Includes ICD-9 diagnoses codes 555,556, 558, and 579

Statistical significance versus no migraine matched controls: ns *P* > 0.05; * *P* ≤ 0.05; ** *P* ≤ 0.01; *** *P* ≤ 0.001

### Pharmacologic treatment

#### Overview of therapies used over the last two years

**Fig. 2:**
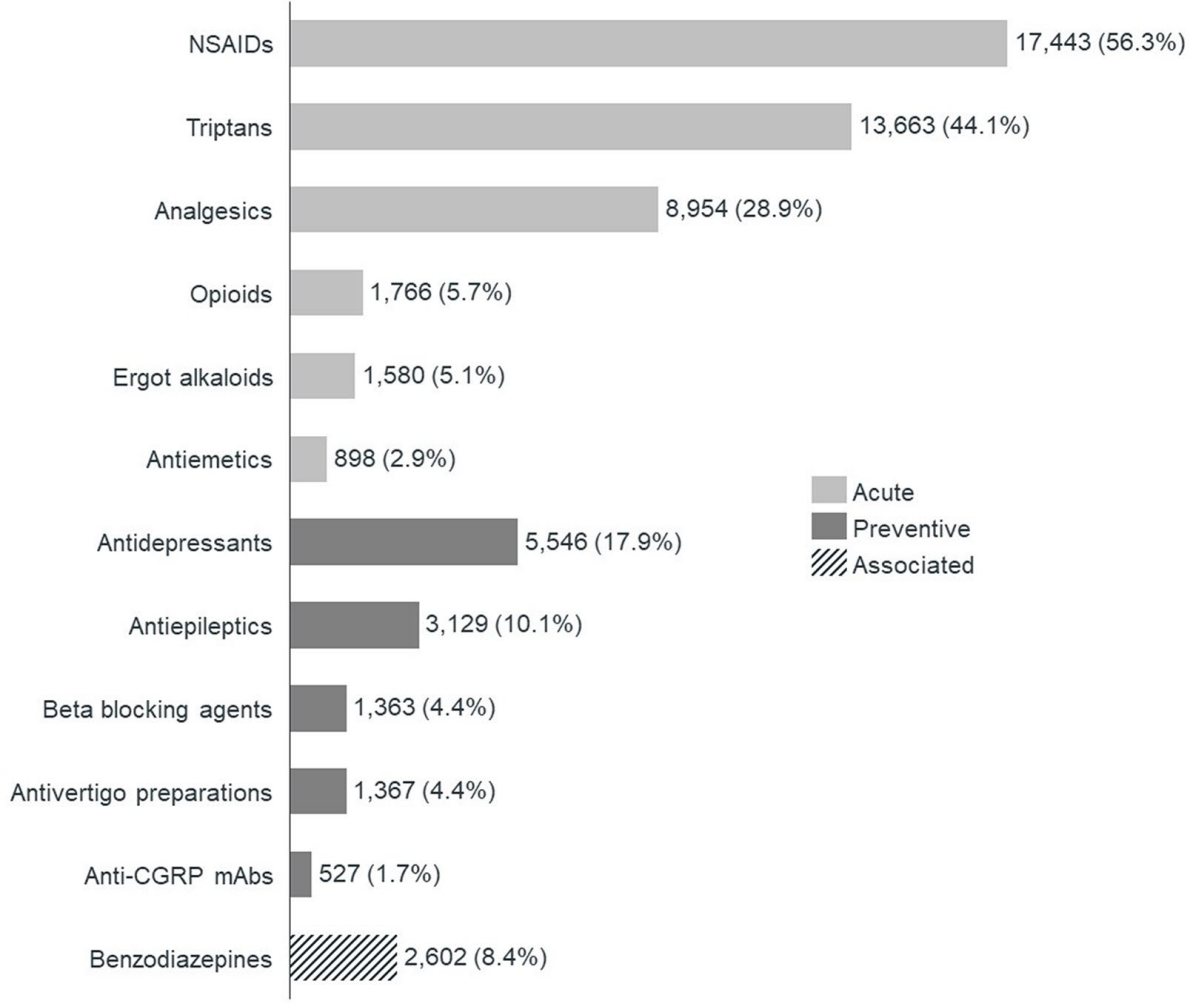
Treatments prescribed to migraine patients from May 2020 to April 2022, per class of treatment, as a percentage of total migraine patients treated from May 2020 to April 2022 (Cohort A). CGRP, Calcitonin gene-related peptide; NSAIDs, Non-steroidal anti-inflammatory drugs. *Note* The percentages add up to more than 100% as patients may have been prescribed more than one class of treatment over the analyzed period

Within the cohort of patients treated for migraine over the last 24 months (Cohort A), 69.5% had been prescribed only acute treatments, 24.2% both acute and preventive treatments, and 6.3% only preventive treatments. The most frequently prescribed treatments were NSAIDs (56.3%), triptans (44.1%) and analgesics (28.9%) (Fig. 2). Antidepressants were the most prescribed preventive treatment and anti-CGRP mAbs the least prescribed. Antidepressants were used by 17.9% of all patients treated over this period and 58.7% of patients treated with a preventive medication. Anti-CGRP mAbs were prescribed to 1.7% of all patients treated over this period and 5.7% of patients treated with a preventive medication. Benzodiazepines were also prescribed to 8.4% of patients who were under preventive or acute treatment.

**Fig. 3:**
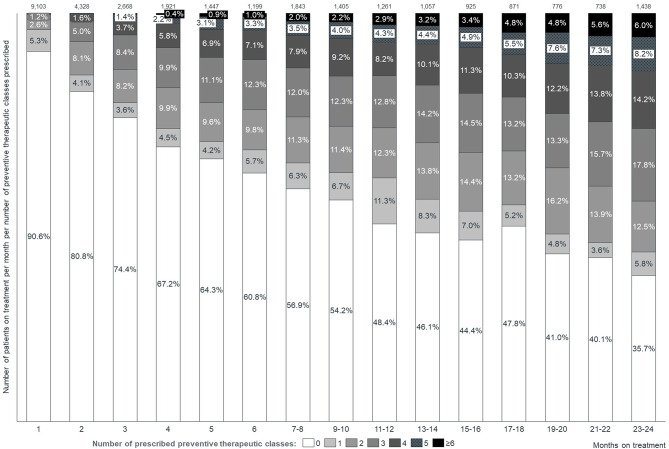
Migraine patients under treatment for migraine per month and evolution of the percentage of patients according to the number received preventive treatments, depending on the length of time under treatment (Cohort A)

In those who had been treated for up to 23–24 months, 35.7% did not receive a prescription for any preventive medication, 5.8% received one, 12.5% two, 17.8% three, and 28.3% received four or more **(**Fig. 3**)**.

#### Choice of initial therapy and progression to preventive treatments

**Fig. 4:**
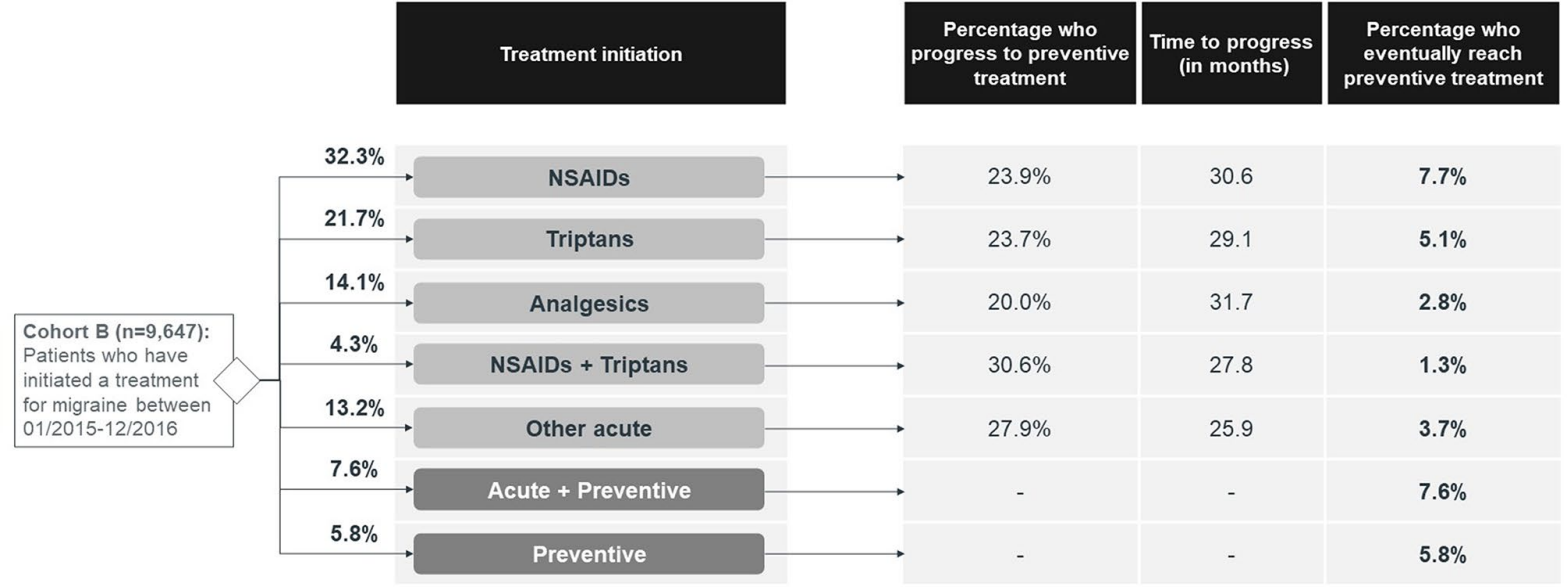
Treatment initiation and progression to preventive treatments during the observation period, amongst physician-diagnosed migraine patients who have initiated a treatment for migraine between January 2015 and December 2016 (Cohort B). NSAIDs, Non-steroidal anti-inflammatory drugs

Most of the patients who initiated a treatment for migraine between January 2015 and December 2016 (Cohort B), had started with a prescription for an acute medication (Fig. 4). NSAIDs alone were selected as the first treatment in 32.3% of cases, triptans in 21.7% and analgesics in 14.1%. Preventive medications were selected as the first migraine prescription treatment in 13.4% of cases: alone in 5.8% of cases and together with an acute medication in 7.6%.

One out of five patients initiating an acute treatment eventually progressed to being prescribed a preventive treatment (20.7%). On average, it took 29.4 months for this change to occur (Fig. 4). In total, a third of the physician-diagnosed migraine had received a preventive treatment until April 2022 (34.0%).

#### Use of anti-CGRP mAbs

**Fig. 5:**
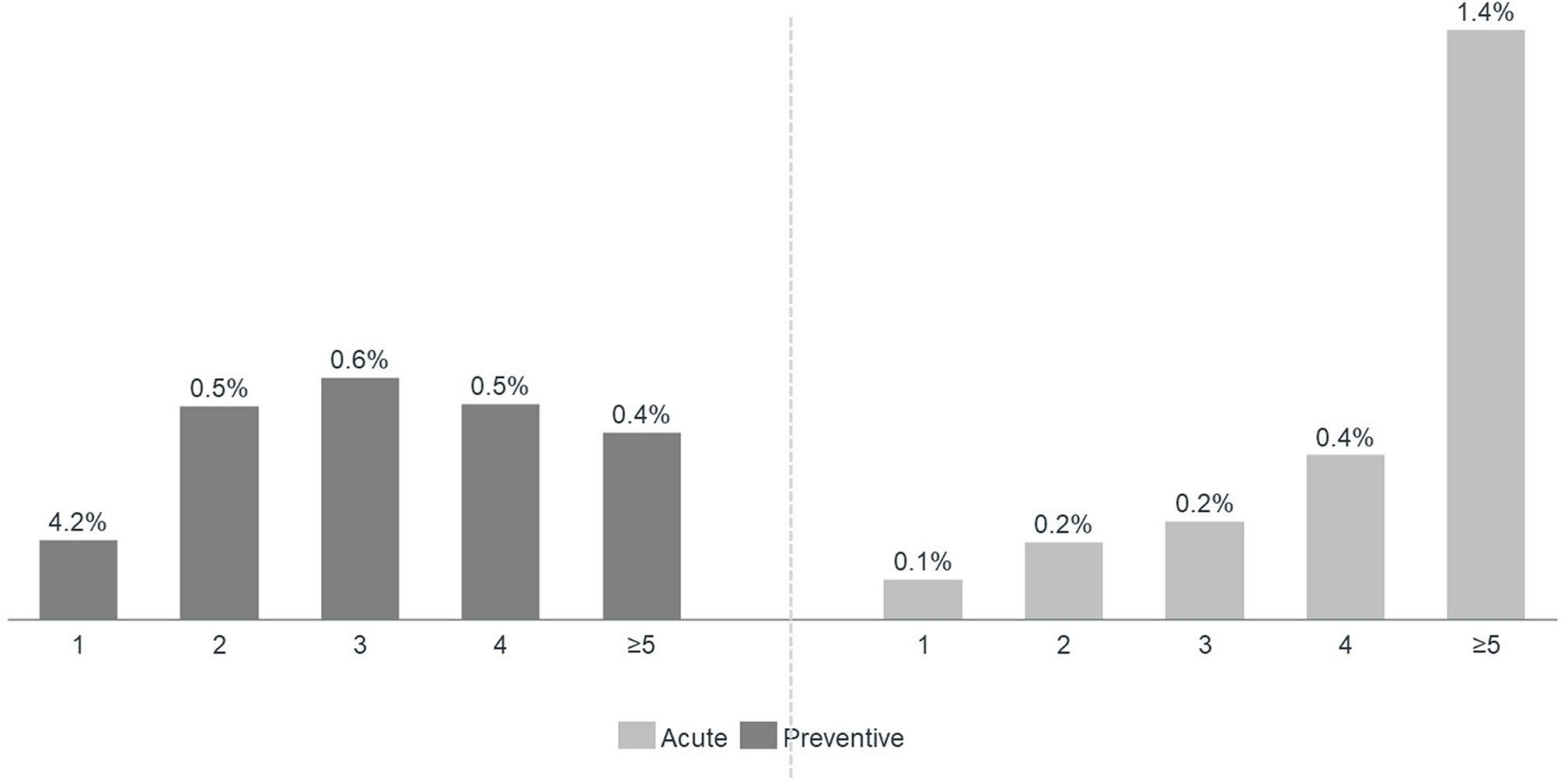
Percentage of migraine patients prescribed with anti-calcitonin gene-related peptide monoclonal antibodies per number of previous acute and preventive treatments received between January 2015 and April 2022 (Cohort F)

Amongst migraine patients who had been prescribed an anti-CGRP mAb (Cohort F), 44.8% received galcanezumab, 43.5% erenumab and 11.8% fremanezumab. Before starting on an anti-CGRP mAb, 66.3% of patients had previously used ≥ 3 conventional preventive and 87.3% had used ≥ 3 acute medications (Fig. 5).

### Disease management

#### Role of GP per patient treatment cohort

**Fig. 6:**
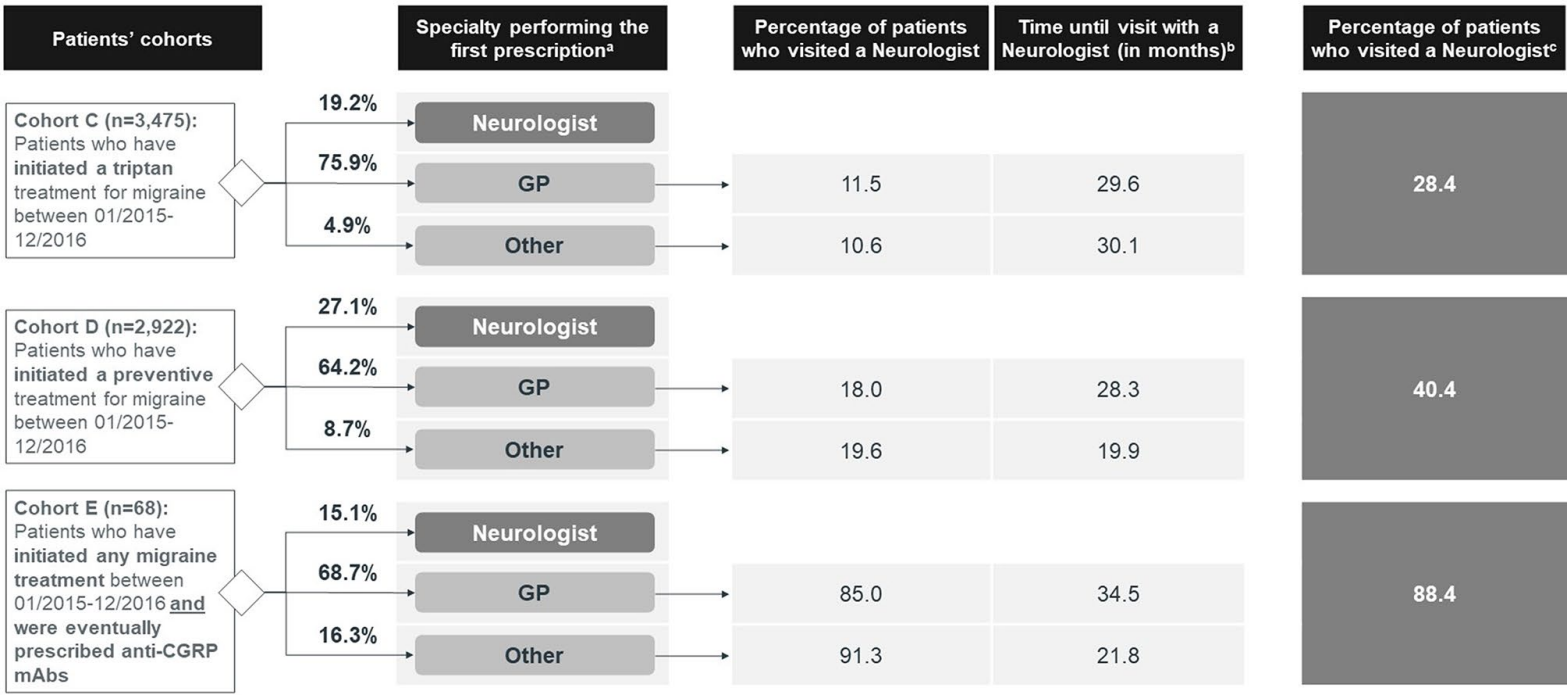
Migraine patients managed by a neurologist per type of treatment initiated between January 2015 and December 2016 and time until referral (Cohorts C, D and E). CGRP, Calcitonin gene-related peptide; GP, General Practitioner. a. Treatments initiated between January 2015 and December 2016. Patients have been followed up since the first analyzed treatment found in the inclusion period until the last month with available data (April 2022). Only the treatments mentioned in the respective cohort have been included in the analyses. b. Time until first visit to the neurologist, regardless of whether there were previous visits to other specialties. c. Analysis performed up until the third visited specialty

Figure 6 illustrates the journey across medical specialties from the first treatment onwards for three patient cohorts (C, D and E). Most patients started their migraine treatment with a GP. Amongst patients who initiated their migraine treatment with triptans (Cohort C), 75.9% were first managed by a GP, 19.2% by a neurologist, and 4.9% by another specialist. An additional 9.2% of patients visited a neurologist, on average, 30 months after the initial prescription by a GP or other specialist. The majority of patients in this cohort (Cohort C) have been first prescribed rizatriptan (40.4%). After more than two years of treatment (average 28.2 months), approximately a quarter of patients switch to another line of triptan treatment (23.2%), as detailed in Supplementary Materials (Figure S2).

Similar pathways were observed for patients who started with a preventive treatment (Cohort D), although with a higher share of cases managed by a neurologist. Amitriptyline was the most common choice for the first preventive treatment prescription (prescribed to 38.2% of patients who started a preventive treatment), followed by topiramate (15.9%), and flunarizine (15.4%). One year later, on average, 31.1%, 40.0% and 39.5% of patients who started with amitriptyline, topiramate, and flunarizine, respectively, had switched to a second line of preventive treatment, as detailed in Supplementary Materials (Figure S3).

Within patients who started with any migraine treatment and who, in due course, were prescribed anti-CGRP mAbs (Cohort E), 15.1% started being managed by a neurologist. All patients have at some point visited a neurologist in a hospital for the anti-CGRP mAbs’ prescription.

#### Role of GP per number of preventive treatments

**Fig. 7:**
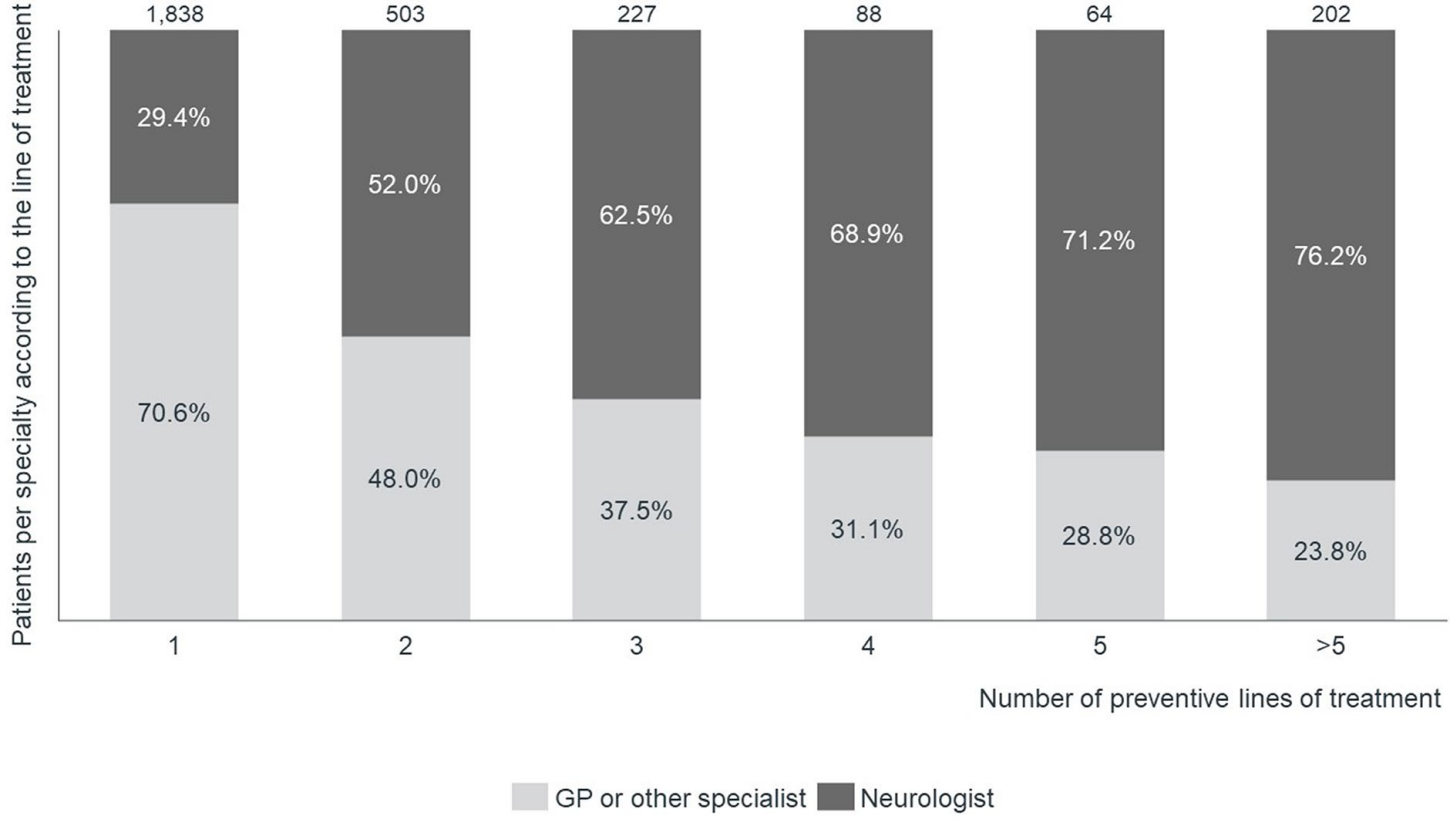
Percentage of migraine patients who have initiated a preventive treatment for migraine between January 2015 and December 2016 who reach a neurologist per number of preventive lines of treatment registered between January 2015 and April 2022 (Cohort D). GP, General Practitioner

Amongst patients who initiated a preventive treatment between January 2015 and December 2016 (Cohort D), the percentage of patients treated by a neurologist increased with the number of received preventive medications (Fig. 7). However, 28.8% of patients who had already been prescribed five or more distinct preventive treatments were not treated by a neurologist.

## Discussion

To our knowledge, this is the first study to analyze treatment algorithms and prescription patterns for migraine treatments across GPs and neurologists in Spain. Our findings highlight a set of unmet needs for improved management of migraine to reduce the risk of chronification and improve patient outcome. We identified risk factors for migraine progression, including multiple associated comorbidities and several lines of treatment.

### Need for improved diagnosis and accurate coding of migraine diagnoses in the healthcare systems

Our study findings indicate a high and increasing number of patients with physician-diagnosed migraine, consistent with published literature [7, 36]. However, our reported prevalence of physician-diagnosed migraine (5.5% of all age population and 6.5% of people aged > 18 years old) is lower than prevalence estimates published for Spain, potentially due to differences in methodologies [36, 37]. Our study only included patients diagnosed with migraine by an NHS physician and required accurate coding of migraine diagnoses in the healthcare systems. In contrast, published estimates of migraine prevalence in Spain are based on population surveys and include undiagnosed migraine cases [36, 37]. Nonetheless, when considering both the diagnoses of migraine and treated headaches, we obtain an estimated prevalence of 12.7%, which is closer to the one published in population-based surveys, suggesting possible diagnostic errors, as reported in other studies [6, 45, 46]. Training to physicians, namely in primary care, may be necessary to improve the diagnosis of migraine [35, 45, 46]. 

### Need for improved coordination of care and appropriate referral patterns to neurologists

This study confirms the central role of primary care in treating migraine patients in Spain – even in difficult-to-treat cases with high prior preventive treatment failure –, as already reported [6, 39]. GPs prescribed the first preventive treatment to 64.2% of patients. Even in those who had already been prescribed five or more distinct preventive treatments, 28.8% were still not being managed by a neurologist. These results may require some reflection. Whilst GPs are advised to manage most migraine patients, complex cases should be referred to neurologists [47]. Patients and healthcare professionals could benefit from a greater clarification of the role of each physician in the management of migraine and on when to refer to a neurologist [35, 45, 46]. 

### Need to address specific needs of patients with associated comorbidities to mitigate risk of progression

As expected, most migraine patients in our study were women of working age with several comorbidities [7, 37, 39]. The most frequently observed comorbidities were coexisting chronic pain (50.8%), metabolic (38.6%), neuropsychiatric (35%), digestive (25.4%), and cardiovascular (25.4%) conditions. These findings support the notion that migraine is frequently associated with other medical conditions, including but not limited to psychiatric disorders [39, 48, 49]. Importantly, our study also found that patients with migraine had a higher prevalence of comorbidities than the general population, even after adjusting for age and gender. It is crucial to consider the presence of comorbidities in the management of migraine, as they may contribute to refractoriness or resistance to prescribed treatments and are a risk factor for progression to CM [40, 50]. Studies have shown that patients with more comorbidities are five times more likely to progress to CM than those with fewer comorbidities [50]. Therefore, it is essential to address these risk factors to prevent disease progression and improve patient outcomes in the management of migraine. This can be achieved by taking a comprehensive approach that considers the patient’s overall health and addressing comorbidities as part of the treatment plan [12, 50–55]. 

### Need to optimize treatment and accelerate access to preventive treatment

A decade ago, a study reported that only a small proportion of migraine patients received specific medications or preventive treatment in Spain, despite high levels of disability [38]. Our study findings suggest that undertreatment of migraine remains an issue, with only half of the patients receiving any treatment for migraine in the past 24 months [6, 39, 46, 56, 57]. In those who were treated, many were prescribed several acute and preventive treatments, suggesting an inadequate control. Possible reasons for suboptimal treatment may include patient preference, tolerability issues, and lack of efficacy [6]. Furthermore, according to studies conducted by Pascual et al. (2020) and Díaz-Insa et al. (2023), there are several other factors that may contribute to suboptimal treatment of migraine in Spain [45, 46, 58]. One such factor is the need for further training of GPs on headache management, particularly regarding the use of preventive treatments, especially considering the advent of new therapeutic options, such as CGRP antagonists or neuromodulation devices [46]. Additionally, undertreatment may be a result of the low rate of referral observed, as preventive treatments are not commonly prescribed by GPs in Spain, and some treatments must be administered in a hospital setting [45, 46]. To address these issues, it is important to provide training on how and when to initiate preventive treatments and establish protocols for the follow-up, treatment, and referral of patients with migraine [35, 45, 46]. 

However, the complexity of migraine management is a concern even in headache centers, as shown by the European BECOME study, which encountered a high proportion of patients who had tried multiple preventive medications and had a history of medication overuse [39]. Improving the management of these difficult-to-treat patients is necessary, as poorly controlled migraine increases the risk of medication overuse and development of MOH and CM, and may contribute to a reduced effectiveness of some preventive treatments [5, 13, 59–61]. 

### Need to address potential barriers in access to innovative treatments

Anti-CGRP mAbs, marketed since the end of 2019 in Spain, were used by 1.7% of migraine patients treated between May 2020 and April 2022, and by 5.7% of those treated with a preventive medication during that period. Patients who were prescribed a CGRP antagonist had already been prescribed a considerable number of preventive and acute therapies: 66.3% had used ≥ 3 preventive and 87.3% had used ≥ 3 acute medications. The number of previously prescribed therapies is expected to be even higher in these patients as the reimbursed indication requires ≥ 3 prior treatment failure, necessarily including BoNT/A for CM, medication which was not captured in our study.

Real world evidence from the Spanish MAB-MIG registry also suggest that patients are reaching anti-CGRP mAbs only after a long time under treatment and after more lines of treatment than those recommended: on average, the 210 migraine patients who had completed at least 12 weeks of erenumab treatment had failed a mean of 7.8 preventive treatments at baseline (including BoNT/A in 95.2% of patients) [17]. 

Possible barriers to access anti-CGRP may include lack of awareness of these novelty treatments, namely in primary care, hospital budget constraints, and overall need to increase the use of preventive medication [35, 46, 58]. 

### Limitations

This study has the standard limitations associated with a database study, such as being subject to coding errors or missing information. Due to the low sensitivity of ICD-9 codes for identifying migraine, the prevalence of migraine is expected to be underestimated in our study [62]. According to a work by Yamato et al. (2023), in which 12 coding algorithms were tested for the identification of people with migraine within a Japanese claims database, the definition used in our study (ICD-9 346) held a high specificity (99.1%) but a very low sensitivity (8.2%). As our goal was to explore the management of migraine patients, the use of this definition minimizes the risk of including false cases of migraine [62]. 

In our study, we encountered limitations related to the segmentation of migraine patients into CM and EM subgroups. Specifically, the regions ICD-9 codification of diagnoses at primary and specialized care did not enable to adequately differentiate patients with chronic vs. episodic migraine. Additionally, the database does not include data on the frequency of headaches. We acknowledge that the lack of differentiation between CM and EM and the lack of patients’ segmentation according to their headaches frequency is an important gap and can affect the readability of some of the results. Additionally, it precluded a detailed analysis of progression from EM to CM over time, which would have provided valuable insights into the impact of current migraine management strategies.

Medication overuse was not assessed in this study due to data limitations. Specifically, the database does not capture data on the number of monthly headache days, nor the number of days of migraine treatment, as only prescriptions are captured. Furthermore, self-medication using OTC products is not captured by the database used in this study.

When assessing the disease management, one must consider that our data does not include prescriptions made by physicians working in the private sector, nor information on whether a prescription made by a GP was supported by a neurologist recommendation. Another limitation is the lack of data on BoNT/A prescription which may result in a data gap when analyzing treatment pathways, particularly in patients treated with anti-CGRP mAbs. To address the fact that some migraine treatments are indicated also for other pathologies, we have considered only the prescriptions with migraine listed as the associated diagnosis.

Importantly, the study period included the COVID-19 pandemic, factor which may have affected the results, especially in the cohort of patients treated in the last two years (between May 2020 and April 2022). It is possible that, without the effect of the COVID-19 pandemic, the rate of migraine diagnosis and treatment could have been higher than the one reported in this study.

Finally, the study uses data from three Spanish regions and may therefore not reflect geographic variations in migraine prevalence rates and treatment practices. The specific regions in the database cannot be disclosed due to confidentiality agreements in place [38]. 

## Conclusions

In short, the study brings to light a set of concerns that should be addressed to reduce the burden of migraine in Spain, highlighting the need to improve the management of complex cases, as patients are currently undergoing several lines of treatment and have several comorbidities, suggesting an inadequate control of migraine. Treatments should be started earlier, and, if they fail, patients should be offered the next option in a short period of time. Otherwise, they risk having a reduced effectiveness of some preventive treatments. The complex nature of migraine demands a better coordination across healthcare professionals for an optimal management, particularly in patients with comorbidities and other risk factors which may lead to refractoriness. A National top-down Migraine Healthcare plan should be implemented to ensure appropriate care across the country.

### Electronic supplementary material

Below is the link to the electronic supplementary material.

Supplementary Material 1

## Data Availability

The data that support the findings of this study are available from IQVIA, but restrictions apply to the availability of these data, which were used under license for the current study, and so are not publicly available. Data are however available from the authors upon reasonable request and with permission of IQVIA. Those wishing to request the data from this study should contact the author Mafalda Carmo.

## Abbreviations

ADHD: Attention–deficit/hyperactivity disorder
AMI: Acute myocardial infarction
AP: Angina pectoris
ATC: Anatomical Therapeutic Chemical
BoNT/A: Botulinum toxin type A
CM: Chronic migraine
CGRP: Calcitonin gene–related peptide
COPD: Chronic obstructive pulmonary disease
EMR: Electronic Medical Records
EM: Episodic migraine
GP: General Practitioner
ICD: 9–International Classification of Diseases 9th Revision
M: Million
mAb: Monoclonal antibody
NHS: National Healthcare Service
NSAIDs: Non–steroidal anti–inflammatory drugs
