# Supplementary material for: Migraine treatment: quo vadis? Real-world data study (2015–2022) in Spain
Source: BMC Neurol. 2024 Apr 2;24:107. doi: 10.1186/s12883-024-03600-8 (PMC10985889; doi:10.1186/s12883-024-03600-8)

### Table S1. Diagnostic codes used to identify analyzed comorbidities

| **Type of Comorbidity** | **Detail of Comorbidity** | **ICD-9 codes** |
| --- | --- | --- |
| Cardiovascular | Acute pulmonary heart diseases | 415-417 |
|  | Chronic cardiac rheumatic disease | 393 - 398 |
|  | Hypertensive Disease | 401-405 |
|  | Ischemic Disease (AMI/AP) | 410-414 |
|  | Other CV diseases | 420-429 |
|  | Peripheral CV Disease | 440-449 |
| Stroke | Stroke | 430-438 |
| Digestive | Gastrointestinal diseases type 1 | 530 - 539, 569 |
|  | Gastrointestinal diseases type 2 | 555, 556, 558, 579 |
| Metabolic | Dyslipidaemia | 272 |
|  | Patients with 30Kg/m2 will be classified as obese* | 278.0, 278.00, 278.01 |
|  | Diabetes (Type 1 / Type 2) | 250 |
|  | Hypothyroidism | 244 |
| Neurologic | Epilepsy | 345 |
| Neuropsychiatric | Anxiety, Dissociative and Somatoform Disorders | 300 |
|  | Bipolar Disorder | 296 |
|  | Depression | 311 |
|  | Drug abuse | 305 |
|  | Drug dependency | 304 |
|  | Other syndromes | 307 (*except 307.4) |
|  | Stress syndromes | 308 |
|  | ADHD | 314 |
| Pain | Chronic Pain, Fibromyalgia, Generalised Pain | 338.2, 338.4, 729.1, 780.96 |
|  | Dorsopathies | 720-724 |
|  | Chronic Fatigue Syndrome | 780.71 |
|  | Disorders of the peripheral nervous system | 350-359, 729.2, 337.0, 337.1, 337.9 |
|  | Osteoarthrosis and related disorders | 715 |
|  | Temporomandibular joint disorders | 524.6 |
| Respiratory | Asthma / COPD | 493 |
|  | Sinusitis | 473 |
| Rheumatologic | Arthritis | 714 |
| Sleep | Organic Sleep disorders | 327 |
|  | Sleep disorders | 307.4 |
|  | Sleep disorders | 780 |

### Table S2. List of pharmacologic treatments included in the analysis

| **Class of treatment** | **ATC code** | **Included molecules** | **Type of use** |
| --- | --- | --- | --- |
| Ergot alkaloids | N02CA | Ergotamine, Dihydroergotamine | Acute |
| Antiemetics | A03F | Metoclopramide, Other Antiemetics (Alizaprida, Cinitapride…) | Acute |
| Benzodiazepines | N05BA | Diazepam, Other Benzodiazepines (Alprazolam, Lorazepam…) | Migraine associated |
| Antivertigo preparations | N07C | Flunarizine, Other Antivertig (Betahistine, Cinnarizine) | Preventive |
| Opioids | N02A | Morphine, Other Opioids (Codeine, Oxycodone…) | Acute |
| Serotonin agonists (Triptans) | N02CC | Almotritptan, Eletriptan, Frovatriptan, Naratriptan, Rizatriptan, Sumatriptan, Zolmitriptan | Acute |
| Calcitonin gene-related peptide antagonists | N02CD | Erenumab, Galcanezumab, Fremanezumab | Preventive |
| Analgesics | N02BA | AAS, Diflunisal, Etersalato, Fosfosal, Salicyloysalicylic acid | Acute |
|  | N02BB | Metamizol, Phenazone, Propyphenazone |  |
|  | N02BE | Paracetamol |  |
|  | N02BG | Clonixinato de Lisina , Nabiximols |  |
|  | B01AC91 | AAS |  |
|  | B01AC06 | AAS |  |
| Non-steroidal anti-inflammatory drugs (NSAIDs) | M01A | Ibuprofen, Naproxen, Dexketoprofen, Other NSAIDs (Acemetacin,Flurbiprofen, Glucosamine…) | Acute |
| Antiepileptics | N03A | Topiramate, Other Antiepileptic (Beclamide, Cannabidiolo, Valproic acid…) | Preventive |
| Antidepressants | N06A | Amitriptyline, Other Antidepressant (Agomelatine, Nortriptyline…) | Preventive |
| Beta blocking agents | C07A | Propranolol, Other Beta - Blockers (Acebutolol, Bisoprolol, Carvedilol…) | Preventive |
|  | C07B | Beta blocking agents with Thiazides |  |
|  | C07C | Beta blocking agents with Diuretics |  |
|  | C07D | Beta blocking agents with Thiazides and Diuretics |  |
|  | C07EA | Other combinations |  |
|  | C07F | Other combinations |  |

### Figure S1. Lines of therapy analysis definitions


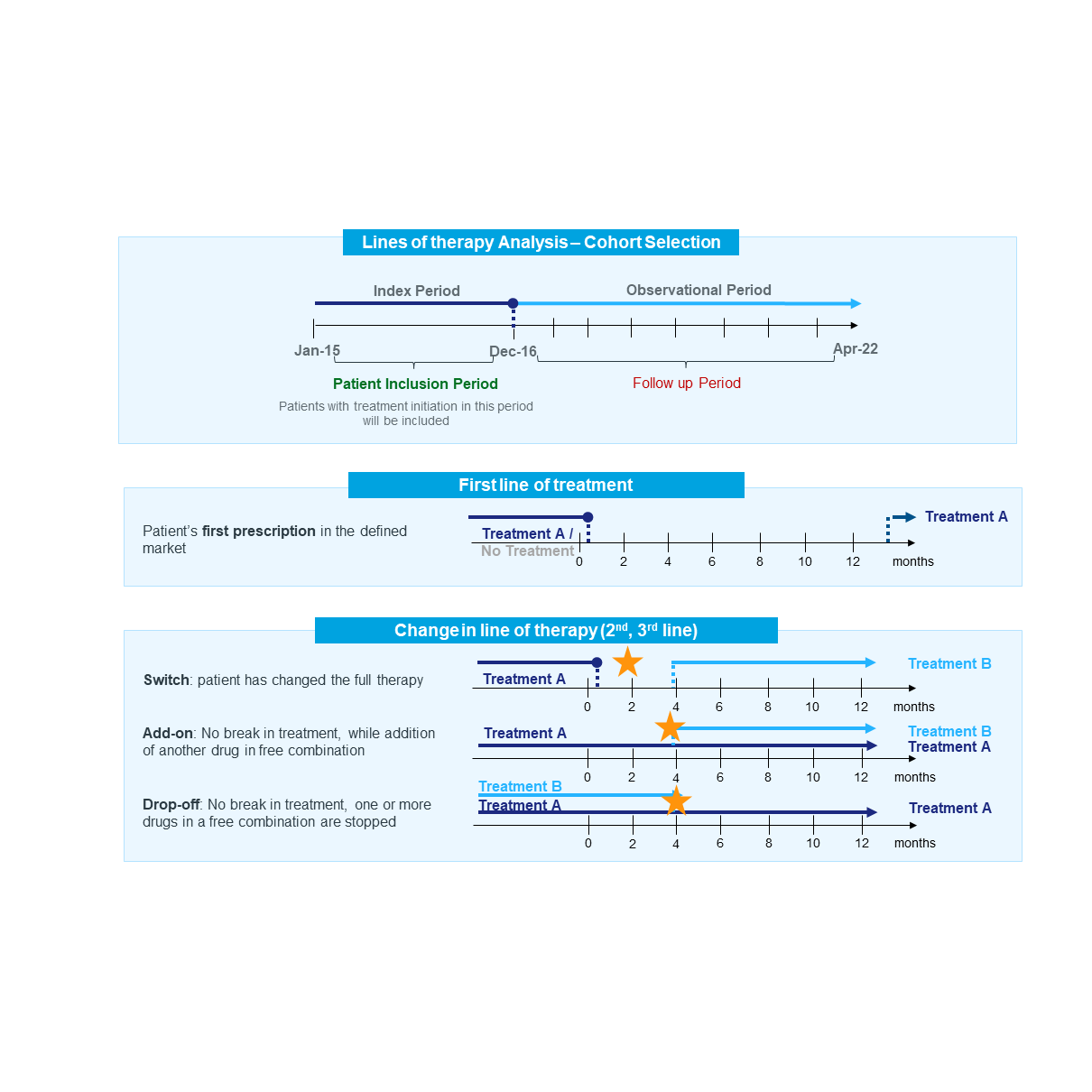


### Figure S2. Lines of therapy analysis per molecule: focus on triptan treatments


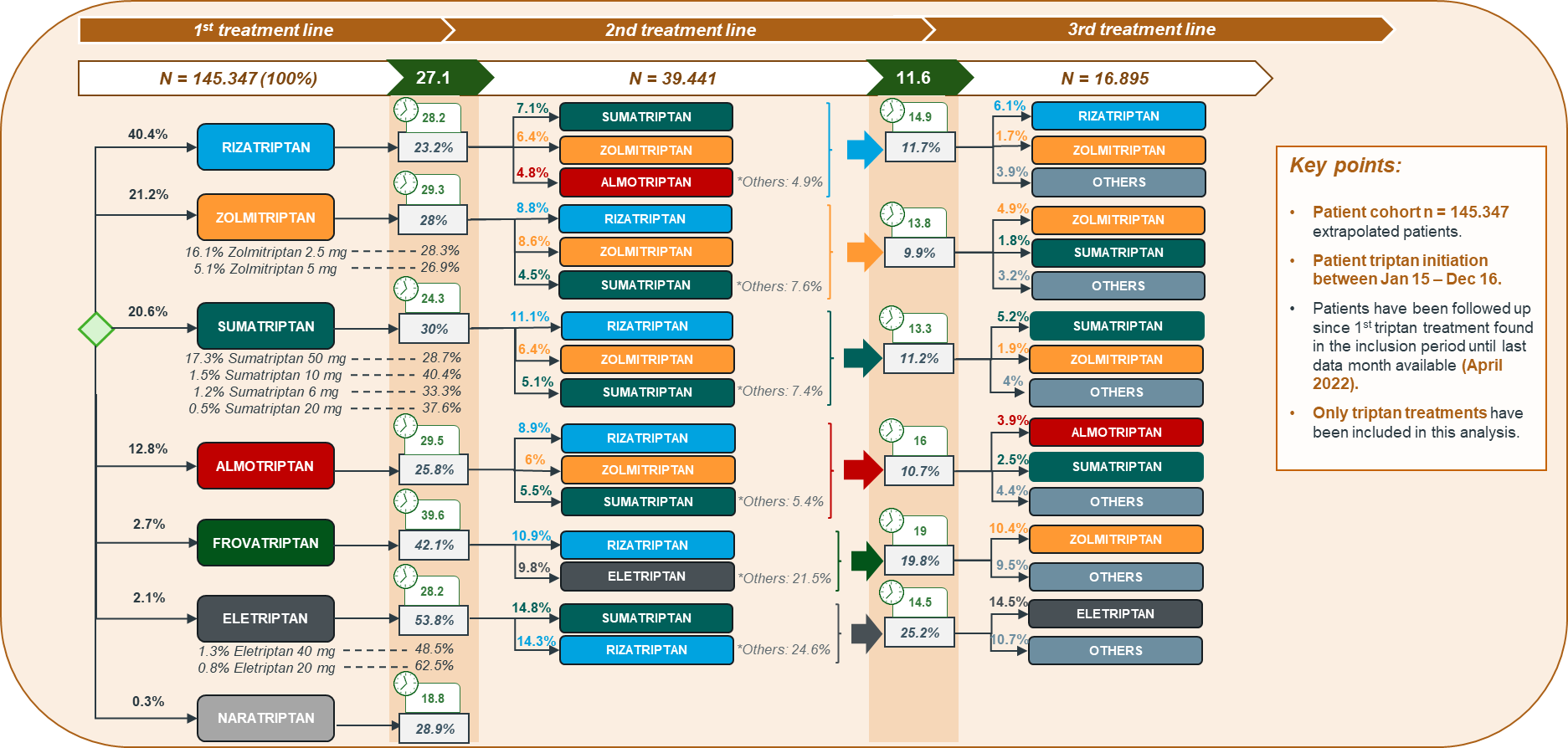


### Figure S3. Lines of therapy analysis per molecule: focus on preventive treatments


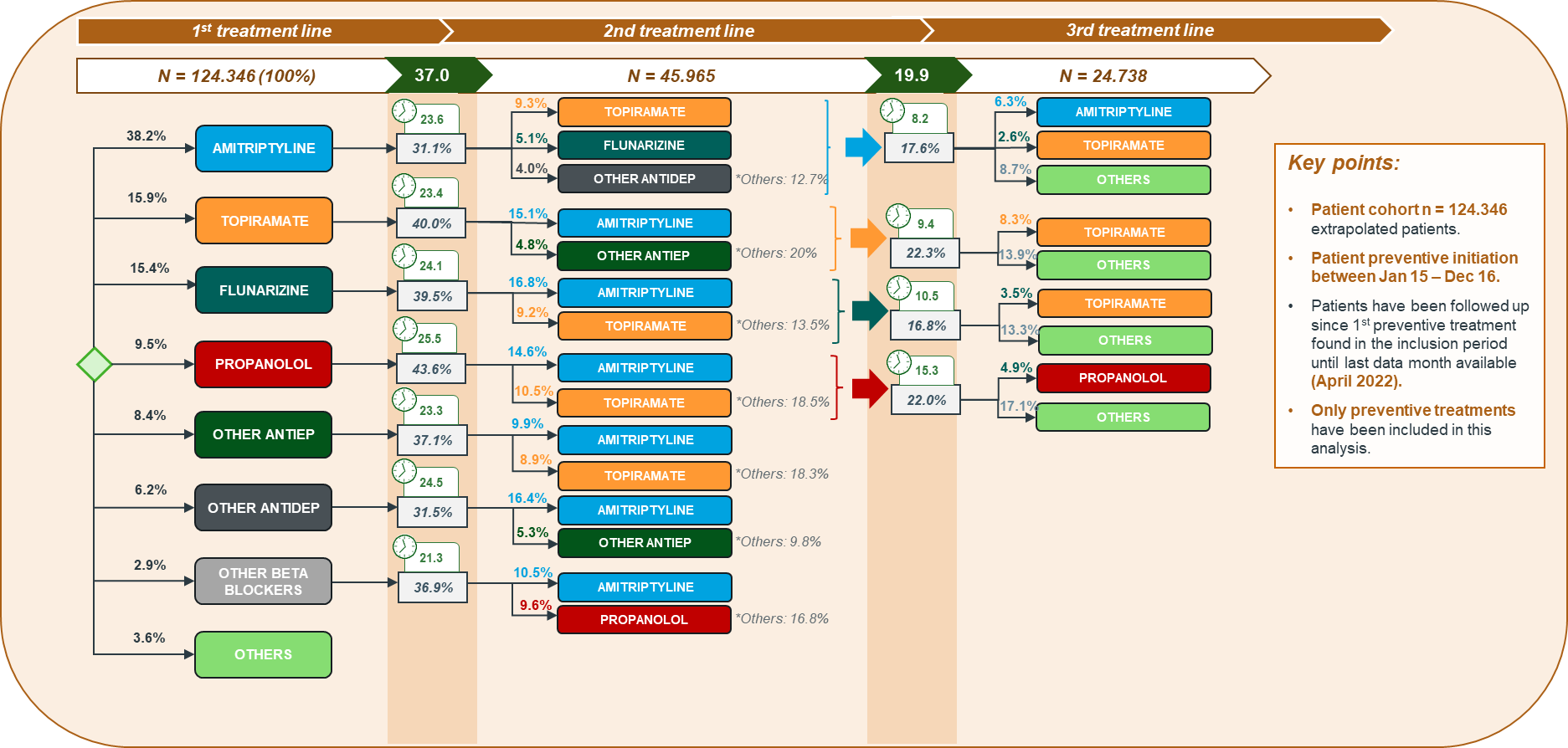


### Figure S4. Detailed patient pathway: focus on triptan treatments

1. All patients who initiated a triptan between January 2015 and December 2016


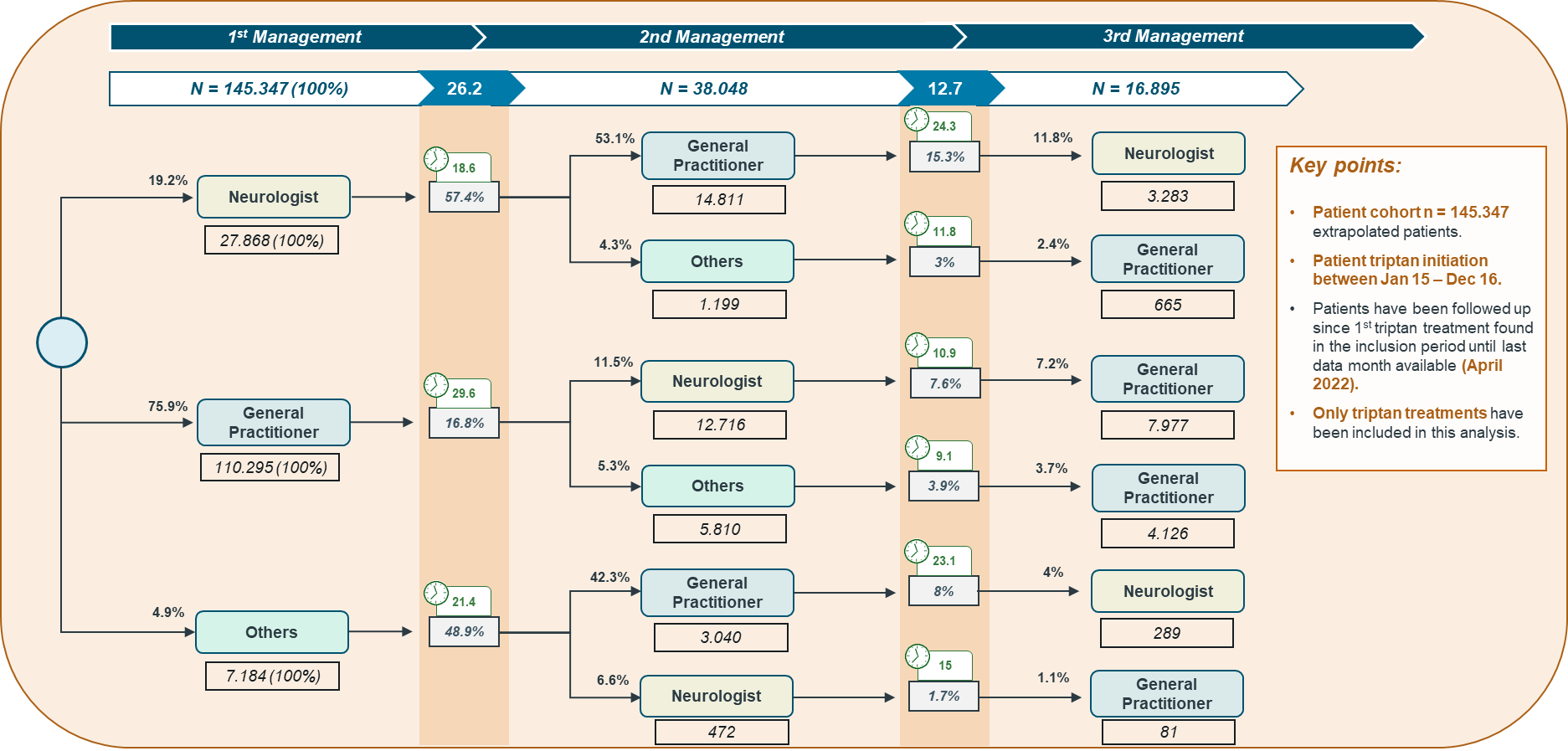


1. Patients who initiated a triptan between January 2015 and December 2016 and with more than two different triptan molecules in their historical record


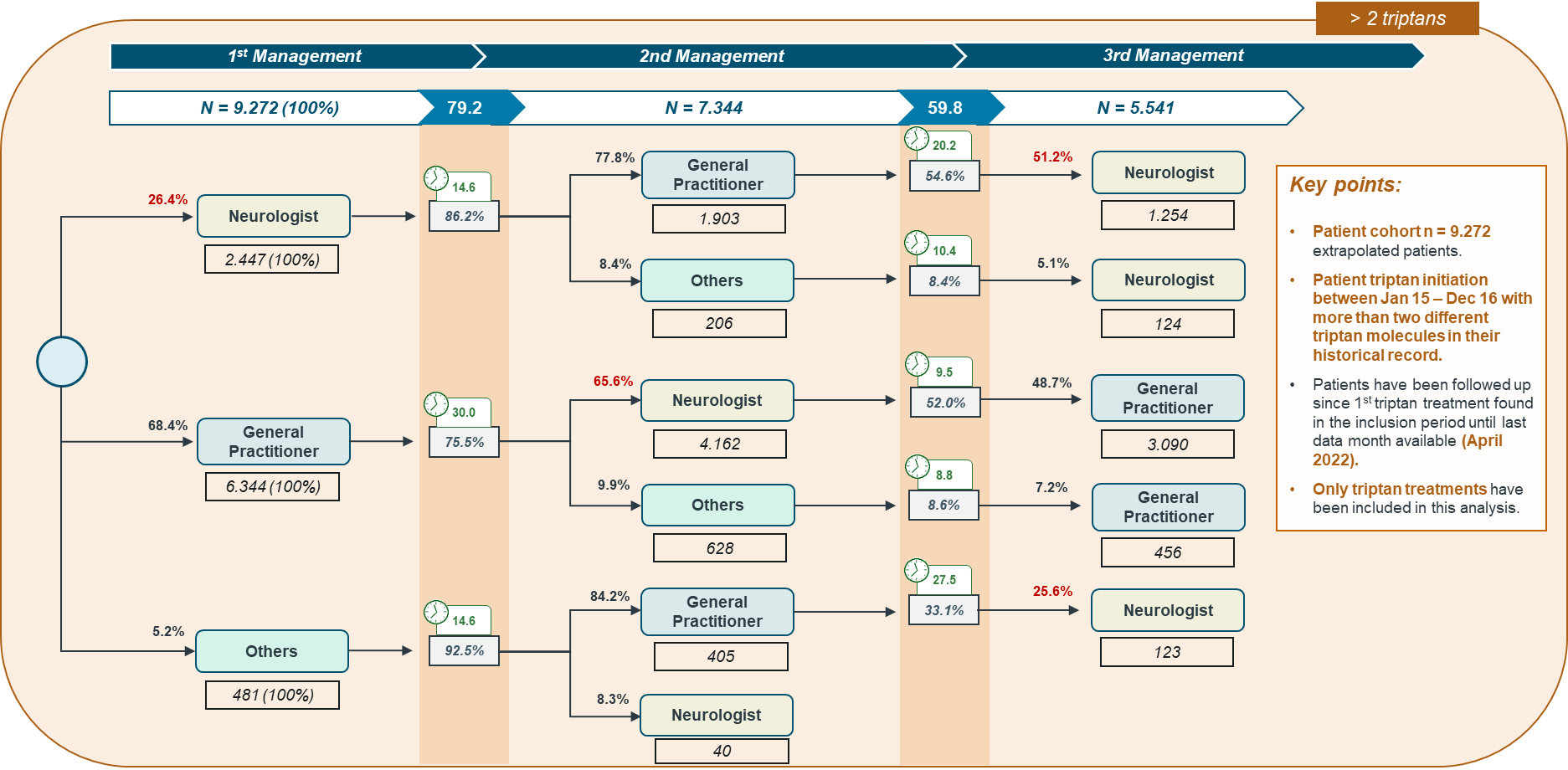

Supplement: Supplementary file 1 — Supplementary Material 1 [file 12883_2024_3600_MOESM1_ESM.docx]
